# Mobilising stakeholders to improve access to state-of-the-art radiotherapy in low- and middle-income countries

**DOI:** 10.3332/ecancer.2021.1227

**Published:** 2021-05-10

**Authors:** Ndimofor Chofor, Pierre Bopda, Rebecca Bücker, Azeh Ivo, Ernest Okonkwo, Kra Joel, Zanzem Tung, Taofeeq Ige, Holger Wirtz, Wilfred Ngwa

**Affiliations:** 1 Strahlentherapie Leer, Annenstr 7, 26789 Leer, Germany; 2 Strahlentherapie Agaplesion Diakonieklinikum Rotenburg, Elise-Averdieck-Str. 17, 27356 Rotenburg, Germany; 3 Strahlentherapie Klinikum Lippe GmbH, Rintelner Straße 85, 32657 Lemgo, Germany; 4 Onkologische Praxis und Tagesklinik, Ahstr. 2, 45879 Gelsenkirchen, Germany; 5 Strahlentherapie Ortenau Klinikum, Weingartenstr. 70, 77654 Offenburg, Germany; 6 Radiotherapy Department, Military Hospital, PO Box 3377, Kigali, Rwanda; 7 Zentrum für Strahlentherapie und Radioonkologie, Mozartstraße 30, 26655 Westerstede, Germany; 8 Medical Physics Department, National Hospital Abuja, Abuja, FCT 900001, Nigeria; 9 University of Abuja, Abuja, Nigeria; 10 Strahlentherapie Singen-Friedrichshafen, Virchowstraße 10b D-78224 Singen/Hohentwiel, Germany; 11 Harvard Medical School, Brigham and Women’s Hospital, Dana Farber Cancer Institute, Boston, MA 02115, USA; 12 University of Massachusetts Lowell, Boston, MA 02115, USA

**Keywords:** radiotherapy access, low- and middle-income countries, information and communication technologies, solar-powered radiotherapy

## Abstract

In an ongoing effort to improve access to state-of-the-art radiotherapy in low- and middle-income countries (LMICs), a joint symposium was organised by the non-governmental, non-profit organisation Medical physicists in diaspora for Africa e.V. (MephidA e.V.) in collaboration with the Germany-based Cameroon-German medical doctor’s association (Camfomedics e.V.) and the Harvard-based Global Health Catalyst summit. The goal of the symposium was to discuss the technical and structural challenges faced in African LMIC settings, re-evaluate strategies to overcome the shortfall of radiotherapy services and ameliorate the situation. The meeting brought together industry partners, including radiotherapy machine vendors and dosimetry solution providers, alongside public health, oncology and medical physics experts. This paper summarises the deliberations and recommendations based on the ongoing efforts including the use of information and communication technologies towards the provision of expert knowledge and telemedicine, the use of solar energy to avoid power outages and the use of high-end technology for enhanced quality assurance. We also present the experiences on the first linac installation at the Rwanda Military Hospital, the challenges faced in this LMIC as well as the patient’s demography, reflecting the reality in most sub-Saharan LMICs.

## Background

Alongside surgery and chemotherapy, radiotherapy has considerably improved over the past decades and plays an important role as one of the major treatment modalities for up to 50% of newly diagnosed cancer cases, thereby serving as a critical component of a comprehensive cancer control programme [1–3]. Furthermore, in palliative care, radiotherapy is a major modality for improving the quality of life of patients [4]. There are ongoing global initiatives to lower the burden of cancer and to increase the access to diagnosis and state-of-the-art radiotherapy treatment, especially in African low- and middle-income countries (LMICs) [5]. Over the past years, the annual Global Health Catalyst (GHC) summit, taking place at Harvard, in Germany and Africa, has been an exemplary platform to bring together stakeholders from the industry, governments, oncology experts and philanthropists, all engaged in increasing access to cancer therapy [6]. The German-based Cameroon-German medical doctor’s association (Camfomedics e.V.) is also engaged in this cause with a major initiative being the establishment of the telemedicine tumour boards to provide expert reviews on oncology cases and exchange with oncology professionals in LMICs. Medical physicists in diaspora for Africa (MephidA e.V.), a non-profit NGO based in Germany, has ever since its creation in 2015 been an active player in coordinating equipment donation and co-organising meetings, amongst others, to promote the use of radiotherapy in LMICs. MephidA e.V. comprises members of diverse professions, majority of whom are certified medical physicists with many years of experience in radiotherapy. In a joint effort initiated by MephidA e.V. in collaboration with the GHC and Camfomedics e.V., we revisited the shortfall of radiotherapy treatment machines in LMICs and brought together stakeholders from the industry, international community and oncology experts in a virtual symposium held on 24 October 2020, which was hosted from Oldenburg, Germany [7]. In this report, we provide a summary of the deliberations from the conference titled ‘Reducing the cancer burden: Technical and structural challenges in low-income countries’, where the major issues hindering the provision and sustainability of state-of-the-art radiotherapy in LMICs were discussed alongside solutions geared towards increasing access to cancer care.

Increasing access to state-of-the-art cancer care in LMICs requires well-coordinated input from key players, including the local government and agencies, industrial partners, expert knowledge and a well-tailored financial plan to ensure sustainability. Given the interdisciplinary construct and complexity of establishing a comprehensive cancer care programme to support the cancer burden in LMICs, the approach in this meeting was to focus on key aspects that, in our opinion, would contribute significantly to increasing access to cancer care. We assessed the use of information and communication technologies (ICTs) to provide second-opinion diagnostic and treatment views as well as revert the brain drain paradigm into brain gain with major engagement of the resource-laden diaspora. We also evaluated energy requirements and the harnessing of renewable energy sources to operate high-power radiotherapy and diagnostic equipment, thereby avoiding machine degradation and downtime. Deliberations at the conference also considered the utilisation of smart and remote-assisted quality assurance (QA) solutions to support ground staff, thereby improving efficiency and avoiding error sources. The conference also considered the use of efficient and modern treatment delivery devices alongside hypofractionation treatment schemes to increase patient throughput, while maintaining the same therapeutic outcome. A case-study example of the successful launch of the radiotherapy department at the Rwanda Military Hospital (RMH) in Kigali, Rwanda, was presented and the typical challenges faced in an LMIC setting were addressed. Furthermore, four leading companies in the field of radiotherapy device applications were represented and reported during the meeting, giving guiding experiences and prospects.

In the concluding panel discussion, five expert panellists presented their views on the necessary prerequisites to set up a comprehensive cancer care centre in an LMIC. The experts included (i) a representative from a leading dosimetry and solutions vendor from the industry, (ii) a global healthcare manager with many years of experience working with and in LMICs, (iii) an experienced oncologist from a high-income setting, (iv) a medical physicist expert from an LMIC and (v) a medical physicist expert from a high-income setting. A consensus drawn from this inclusive discussion was to create partnerships among stakeholders, while highlighting the fact that enabling sustainable radiotherapy access requires the cooperation with local governments and consulting agencies such as the International Atomic Energy Agency (IAEA).

## Shortfall of radiotherapy in LMICs: technical and structural challenges

It should be noted that 57% of cancer cases worldwide occur in LMICs [8, 9]. More than 50% of these patients requiring radiotherapy lack access to treatment. Up to 70% of new cancer occurrences are anticipated in LMICs by the year 2040 [10]. The technical and structural challenges hindering the availability of radiotherapy machines and ensuring the sustainability of cancer care provision have been extensively published [11–13]. Briefly, the combination of a country’s socio-economic health and the regional geo-stability affects the success of cancer care programmes. Although the deliberations in this symposium focused only on improving access to radiotherapy with high-energy linear electron accelerators (linacs), one must mention the potential of brachytherapy to significantly reduce the cancer burden, especially in the case of gynaecological pathologies. Using published data from the Directory of Radiotherapy Centers (DIRAC) database [14] and from correspondence with leading radiotherapy manufacturers: Elekta and Varian, the linac count increased from 396 to 406 between February and October 2020, with new acquisitions of one linac each in Ethiopia, Ghana, Kenya and Togo (first linac), two in Sudan and six in South Africa. From the reports, the present count is that 29 African countries out of 54 now have made investments in modern, state-of-the-art radiotherapy treatment machines to serve their population, with numerous replacements of outdated cobalt-60 devices. Nevertheless, given the increased average life expectancy and economic-related lifestyle changes, the rate of new linac installations is still far below that required to cover the protracted increase in cancer burden in Africa.

The lack of infrastructure to ease the accessibility of treatment centres via well-maintained roads and safe and reliable transportation services also poses a huge issue. Power outages are quite detrimental and may shorten the lifetime of electrical equipment, eventually resulting in permanent damage. Moreover, the lack of certified and qualified personnel to operate and maintain the linacs and associated equipment is one of the biggest problems faced by most LMICs. These two last factors have indeed been repeatedly highlighted by professionals in LMICs, indicating the need for local investment in human resources. Ranging from the unavailability of accredited programmes to train professionals to the relatively low wages on the job, there is an ongoing brain drain to more attractive countries [15]. The upfront investment costs in setting up the required infrastructure and purchasing the linac also pose as a huge hindrance [16]. This has motivated investigations to design more robust and affordable linacs for use under circumstantial and environmentally challenging conditions such as during power fallouts and places with high humidity [17].

## Approaches for closing the gap

### Knowledge transfer with ICTs

The multidisciplinary approach in decision-making is regarded as a gold standard in the diagnosis, classification and treatment of almost all cancers and is mandatory in most countries [18]. This multidisciplinary approach has clearly shown its association with improved survival and reduced variations in treatment outcomes [19–21]. Specialties typically involved are the medical oncologist, medical physicist, surgeon, pathologist, radiologist, clinical pharmacist, radiation oncologist, specialist nurse, psychologist and perhaps a social worker. The responsibility of this multidisciplinary team is to make sure all the necessary information are available for cases to be discussed perniciously, to be able to agree and confirm treatment schemes or debate on alternative options. Of course, cases that are more complex may warrant re-evaluation. Owing to recent industrial advances, ICTs are gaining ground in cancer diagnosis and treatment [6, 22, 23]. ICT platforms serve a multipurpose of reversing the brain drain paradigm faced by many LMICs, as well as providing a portal for aspiring professionals in LMICs to access training and certification courses. Most importantly, the potential to leverage the experience of medical practitioners from high-resource countries has a huge benefit, as can be found in the web-based platform for clinical QA [24, 25]. Another exemplary effort is the IAEA-pioneered telemedicine project, Africa Radiation Oncology Network, which was created with the same goal of exposing oncologists and medical physicists in LMICs to expert knowledge [26].

Motivated by the increased number of cancer cases at the Mbingo Baptist Hospital (MBH) in Cameroon and the dearth of specialists in African LMICs, the Internet-based multidisciplinary tumour board (iMDTB) was created. It is hosted by Camfomedics e.V. and run in collaboration with colleagues from Cameroon, the USA and Germany. Using English as the communication language, the board discusses cancer cases and provides state-of-the-art treatment recommendations tailored to the affordability in the setting in Cameroon, in accordance with international guidelines [27]. The iMDTB holds meetings once a month on the first Monday, which last for 60–90 minutes. Since its creation in 2018, more than 200 cases have been discussed, the majority being female. The pathologies involved are breast, cervical, gastrointestinal malignancies, prostate, sarcomas, lung, melanomas, multiple myeloma and head and neck cancers. Most of the patients unfortunately present at advanced stages of the diseases, i.e. non-resectable or with distant metastasis. Thanks to the exchange and discussions by the tumour board, there have been some modifications in certain treatment modalities as a result of extensive histopathological results. For example, in the case of breast cancer, an evaluation of the hormonal status has been introduced to assist further decision-making. This direct consultation with experienced colleagues and specialists has helped reduce the burden of the patients already waiting in line [28, 29]. Although the iMDTB is at present in collaboration with Cameroon, other LMICs are welcome on board, thus in accordance with the motto: best treatment for every cancer patient anywhere on the globe. The rising number of cancer cases and the complexity of the pathologies seen in LMICs reiterate the importance of the iMDTB, as reported in other studies [18, 30]. The benefits of such a multidisciplinary tumour board are manifold. They include:

Medical educational initiatives to highlight awareness activities as keys to encouraging the cooperation between specialties to improve patients’ outcome;

Establishing combined cancer clinics and formal referral systems to encourage a culture of effective communication, joining forces with professionals in peripheral areas and the private sector;

Highlighting the importance of follow-up so that patients feel safe and assured in the course of the disease.

In the iMDTB, we advocate a good relationship between team members, which is important for smooth and effective functioning. There is still a need for improvement to circumvent some problems, which may hinder the overall efficiency of an iMDTB platform. Given the shortage of workers, extra time needs to be allocated towards the preparation and entry of patient data into the digital database. Furthermore, and most often, the decisions made at the tumour board in accordance with international standards cannot be implemented due to several reasons. These are most often due to the unavailability of resources and ineffective management of resources, if available. Based on our experience presented at the conference, this approach presents a powerful model for involving the diaspora in teleoncology, especially for Africa.

### Power fallouts and renewable energy solutions

Owing to their versatility and large range of applicability, modern linacs are preferred for cancer treatment in comparison to cobalt-60 units, a result being an increased electric energy requirement to run the linacs. Given the complex internal computer architecture and network requirements to ensure a seamless provision of end-to-end diagnosis and treatment, power outages can be detrimental to the system unless these components are secured by uninterruptable power supplies. These power supplies are most often huge battery energy storage systems (ESS), which are charged constantly and run in a standby mode, immediately taking over power supply when the grid electric power is unavailable. For dimensioning the required storage capacity of the ESS, it is essential to know the clinic’s workload by day and night. A department in a high-income setting comprising two linacs, one CT and peripheral devices has a typical daytime consumption of about 38 kW and this may drop to as low as one-third at night [31]. Most radiotherapy departments in LMICs operate in two or three shifts, thereby maintaining high energy consumption way beyond daytime energy production. A department in a LMIC with one linac, one CT and one MR unit has an average daily consumption of about 80–100 kW [27].

**Figure 1.**
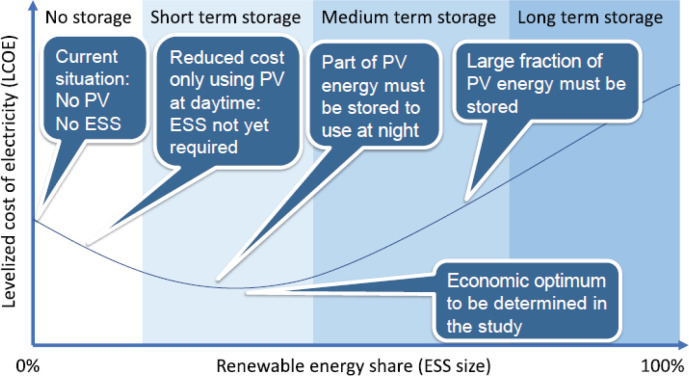
Levelized cost of electricity in relation to the PV generator/ESS combination, indicating the energy consumption target [26]. Figure reproduced with permission from Ralf Bernhard, Deutsche Gesellschaft für Internationale Zusammenarbeit GmbH.

Photovoltaic (PV) systems provide a cost-effective option of covering the energy requirements of a radiotherapy clinic, thus reducing the cost of electricity purchase from the grid or running costs with a diesel generator. The cost of running a PV system alone in Africa is shown to be commercially effective and is further optimised with an ESS option for intermediate energy storage. A proper evaluation of the economic feasibility must therefore be carried out for the local setting. The economic optimum is achieved, for instance, by evaluating the levelised cost of electricity for any given country (see Figure 1). The so-called load study must be tailored to allow scalability to cover further power requirements in the future. The evaluation entails the assessment of the energy autonomy ratio (AR), which is a ratio of the locally used energy to the locally produced energy. This means a 0% AR is achieved for grid-alone dependence, while a 100% AR refers to complete consumption of generated electricity. A limiting factor for this renewable energy option may be the upfront cost of purchasing the PV and ESS, with typical lifetimes of 20 and 10 years, respectively. Vendors of PV/ESS systems offer the possibility to pay off the devices over a variable length of time, depending on the total upfront cost and PV/ESS combination. These requirements are very challenging to meet up with, since many sub-Saharan African countries still battle with the need for providing basic electricity to their communities. Thousands of tons of carbon dioxide emissions shall, however, be reduced over the lifetime of operating the PV/ESS system, thereby showing its environmental and health benefits. The clinic shall also benefit from less equipment damage and longevity of use, as opposed to operation under sporadic grid outages. Another off-grid electricity solution, which is currently being installed at the MBH in Cameroon, is hydroelectricity. The hospital is taking advantage of its location in the waterfall mountainous region of north-west Cameroon. Meanwhile, the plant is anticipated to produce 120 kW of power, and the excess electricity shall be supplied to surrounding neighbours.

### Modern radiotherapy linacs and advanced treatment schemes

Nowadays, most cobalt-60 installations in LMICs are being replaced by modern linacs, capable of fast intensity-modulated dose deliveries and escalating the target dose [32]. These linacs offer numerous advantages over Cobalt-60 machines like delivering higher energies (6–20 MeV) and enabling the treatment of deep tumour sites with considerably improved sparing of organs at risk. Moreover, recent linacs with modern treatment heads are increasingly being available with better multileaf-collimator (MLC) conformance to the treatment volume. Fast MLC speeds and flattening filter-free treatment options with higher dose rates provide the possibility to even further reduce the treatment time by factor 2–4 [33]. In hypofractionated radiotherapy, a higher dose per fraction is delivered to reduce the overall treatment duration by up to 40% compared to classical fractionation schemes. Especially with the present Coronavirus disease (COVID-19) pandemic and the logistic concerns with respect to patient management, there is ongoing evaluation of the potential of hypofractionation to reduce the COVID virus contamination risk [34]. The reduced overall time spent in the clinic effectively protects oncology patients and radiotherapy staff from infection. It has been shown in various studies that the hypofractionated treatment outcomes are comparable to the conventional fractionated schemes with isoeffective sparing of healthy tissues [35]. All these improvements can be introduced in the African/LMIC context without undermining the importance of the precision in the treatment delivery. In a recent publication by the ESTRO-HERO task group 3 on modelling the cost requirements for external beam radiotherapy (EBRT) [36], hypofractionation was shown to reduce the pressure on treatment delivery personnel by up to 27%. For the patients, shortened treatment schemes go a long way to increase throughput, thus benefiting a large number of patients waiting in line for therapy. Recent work at Harvard and collaborating institutions in Africa has demonstrated that hypofractionation can substantially increase access to radiotherapy and save billions of dollars [37]. This approach has been highly recommended for the COVID-19 era and beyond for Africa [38] and was highly recommended coming out of the conference. Keeping in mind that most patients in sub-Saharan LMICs present in late disease stages, there is a need for clinical studies to validate the safe use of hypofractionation under these settings. The implementation of image-guided radiotherapy as an optional step has the potential of increasing treatment delivery quality, but may on the contrary require more treatment delivery personnel. This indicates the need for a thorough assessment of the available technology prior to its implementation, trimming to optimise the usage according to the local cancer burden. Linac vendors at their end give customers the option to make long-term service contracts, with the advantage that well-maintained machines enhance treatment quality with improved sustainability of treatment services.

### QA and LMICs: investing in state-of-the-art technology

Despite the large upfront investment costs of setting up a radiotherapy department, there is a huge economic benefit of acquiring state-of-the-art equipment to assure the quality of the treatment delivery. Investment in modern technology and QA equipment is, therefore, a minimum requirement for sustaining and managing a cancer care programme. Despite the increased trend of investing in modern radiotherapy linacs as opposed to cobalt-60 machines, there remains a challenge of effective usage of this technology without compromising the quality of patient care. This is especially a huge challenge in LMICs for reasons such as the lack of qualified staff, national guidelines, required equipment and a high patient influx [39]. Especially, the lack of recognition of the medical physics profession in several countries has a major drawback on QA, given that certified and well-trained medical physicists are the backbone of QA in a radiotherapy department [40].

In order to solve the challenges faced by allocating time for QA under circumstances of limited staff and material resources, several companies provide QA tools requiring minimal intervention and seamless implementation. For instance, a record-and-verify system to constantly monitor beam delivery does not only enhance the treatment quality, but also has the capability to detect pending technical faults on the linac, with the potential of reducing machine downtime. Nowadays, there are portal dosimetry solutions and offline independent dose computation and verification tools to assure correct dose delivery, but they are insufficient to warrant overall treatment quality. A QA scheme based on the application of risk analysis throughout the entire treatment chain may lead to a more efficient and optimised use of the available resources to obtain the desired therapeutic outcome [41].

### A successful case study: radiotherapy in RMH

The radiotherapy department of the RMH was inaugurated in January 2020 by President Paul Kagame of Rwanda. Two linacs from ELEKTA AB, Stockholm are currently installed. Both linacs are equipped with 160 dynamic multileaf collimators (Agility) and the electronic portal imaging device system, while one has an additional ConeBeam CT imager. Since November 2018, over 682 patients have been treated with the volume-modulated arc therapy technique planned using the Pinnacle treatment planning system version 16.2.1 (Fa. Philips). All radiotherapy plan verifications are carried out prior to treatment, using the Octavius 4D system and evaluated with VeriSoft software (both from PTW Freiburg). Table 1 shows the pathologies treated from January 2019 to October 2020 alongside their total numbers and the number of newly diagnosed cases. These pathologies are common among sub-Saharan LMICs, with patients presenting in late stages of the disease and correspondingly over 90% of patients undergoing palliative treatment [42].

**Figure 2.**
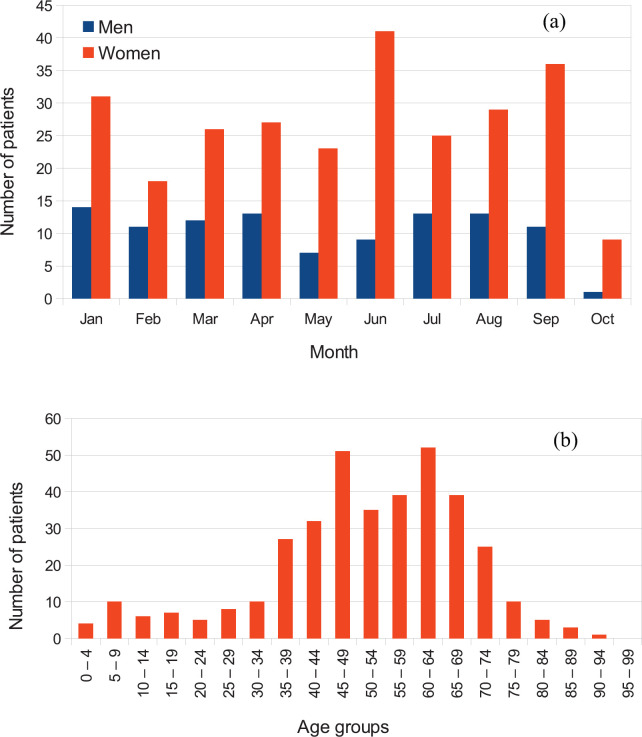
Example of patient distribution by (a) gender and (b) age for patients treated at the RMH from January 2020 until October 2020. Out of 369 treated patients, 104 were male and 265 female. Reported data for October represent only a fraction of the month at the time of data collection.

Being the first two radiotherapy machines in Rwanda, they serve a population of 12 million inhabitants and operate on a working day basis from 8 am to 5 pm. No brachytherapy machine is available. Figure 2a shows the patient demography, indicating distribution by gender, while the distribution by age is shown in Figure 2b. For a total number of 369 patients treated from January 2020 to October 2020 (104 male and 265 female), a significantly higher incidence was recorded in women, an effect common in LMICs and attributable to inadequate access to early detection and treatment [43].

There exists a national cancer control plan in Rwanda since February 2020. Given that the RMH is the first radiotherapy centre in Rwanda, it was a challenging task to convince policymakers on the required budget, project scale and radiation safety. There is a lack of experienced and qualified personnel, requiring that the current government procurement policies need to be adapted to combat this deficit. At present, the department has five clinical oncologists, five radiotherapy therapist technicians, one medical physicist and one technician. Two medical physicists are currently being trained in Morocco under the IAEA programme and are planned to join the physics team in 2021. Although the radiotherapy health insurance covers 90% of treatment costs, most patients still cannot afford the remaining 10%. Additionally, there is insufficient infrastructure to transport and accommodate patients, who often travel from afar for therapy and end up camping in the vicinity of the hospital. The longest time patients spent travelling to the clinic is 6 hours. A diesel-powered generator and UPS systems are available to tackle power outages. Despite the efforts made to get the radiotherapy department up and running, a major challenge still faced is the inefficiency of custom clearance of spare parts delivered from linac manufacturers, with the potential consequence of increased machine downtime. While the industrial partners are collaborating with locals to solve these issues, the current solution has been a local spare part storage at the RMH. The RMH recently signed a long-term service contract with the linac manufacturer, which is a huge step towards sustaining the integrity of the treatment units and maintaining the overall quality of treatment delivery. This project owes its success to the readiness of the government to invest in modern radiotherapy technology, consulting with multinational experts on the requirements for providing the best treatment solutions for their population as well as on mechanisms to ensure sustainability. It is a model to be emulated by other African countries.

### Discussion

With the acknowledgment that Africa remains the most underserved continent in terms of cancer care, some valuable proposals have resulted from our multidisciplinary discussion. In the panel discussion, it was conclusive that a venture to set up a radiotherapy department should include industry representatives, national authorities, regulatory bodies, oncologists, medical physicists and technical advisors for a seamless and sustainable planning process. The planning process would also need to tackle major drawbacks faced in LMICs, such as on strategies to train and provide long-term employment for qualified staff. The decision-making should also take into account the expertise and guidelines from the IAEA on effective implementation of new technology [44, 45]. This is a critical step towards planning the required budget for the upfront and subsequent investments. It was common consensus that a plan to enhance access to high-quality treatment in an LMIC should start with local and affordable resources by:

- Building up regional community cancer care centres for local patient referrals. Also, getting local communities and civil society organisations involved as early as possible and as such bringing back cancer care to the community level.
- Developing strategies to overcome ‘alienation’ of the facility from society, easing access to services and elevating the culture of healthcare. Using understandable and local language, reducing academia bureaucracy and improving infrastructure, especially in palliative care access.
- Taking advantage of the rise of digital health technology, which has a huge potential to change the face of medicine: from artificial intelligence and analytics to drones and telemedicine to expand access and make the provision of healthcare more equitable. With the implementation of artificial intelligence and automated QA tools, some processes such as target definition and treatment planning may help reduce the workload and thereby staff requirement in a radiotherapy department.
- Strengthening the capacity for conducting basic research and clinical trials. The insights gained from these and other initiatives should be translated into effective interventions. A potential outcome would be the tailoring of treatment schemes to the local cancer burden. For instance, the feasibility of hypofractionated dose deliveries in LMIC settings has the potential of drastically increasing the access to care for many patients who wait in line with conventional fractionation schemes.
- Enhancing the procurement of new medicines through secure regulatory harmonisation. Tackling supply chain challenges, improving logistical efficiency and eliminating barriers that have historically prevented medicines from reaching remote and underserved populations.

## Conclusion

Considering the vast amount of revenue spent by cancer patients from LMICs for treatment overseas, increasing radiotherapy access in LMICs would influence local economic growth with potential investment possibilities in transport and logistics facilities, as well as in infrastructure for patients undergoing treatment. Meanwhile, innovative treatment cost subvention schemes need to be developed and integrated into national cancer care programmes; healthcare management in general must also make it a priority to bring innovative solutions into the healthcare system. The plan to successfully provide sustainable radiotherapy access should be case-specific to support the country’s cancer burden and cost-effectiveness with regard to the choice of linac, keeping in mind the upfront, running and maintenance expenses. This venture must, therefore, involve the local governments and consulting agencies such as the IAEA as well as the industrial partners. This forms the basis for the structural organisation of any prospective cancer care programme. We thereby address the role of these cooperating organisations and other stakeholders in a joint advocacy towards expert knowledge transfer to LMICs. All of these efforts may fail if there are no supporting policies and regulations to assure sustainability and upscaling of services.

## Conflicts of interest

There is no conflict of interest.

## Figures and Tables

**Table 1.** Pathologies treated at the RMH from January 2019 until October 2020, comprising of 552 patients, 514 of whom were newly diagnosed cases.

Pathologies	Number of cases, *n* = 552 (%)	Number of new cases, *n* = 514
Gynecology	206 (37.3)	186
Breast	86 (15.6)	84
Head and neck	67 (12.1)	64
Prostate	62 (11.2)	58
Gastrointestinal	60 (10.9)	57
Others	29 (5.3)	26
Neurology	23 (4.2)	21
Thorax	10 (1.8)	10
Skin	7 (1.3)	6
Benign	1 (0.2)	1
Lymphoma	1 (0.2)	1
